# The Entner-Doudoroff and Nonoxidative Pentose Phosphate Pathways Bypass Glycolysis and the Oxidative Pentose Phosphate Pathway in Ralstonia solanacearum

**DOI:** 10.1128/mSystems.00091-20

**Published:** 2020-03-10

**Authors:** Poonam Jyoti, Manu Shree, Chandrakant Joshi, Tulika Prakash, Suvendra Kumar Ray, Siddhartha Sankar Satapathy, Shyam Kumar Masakapalli

**Affiliations:** a BioX Center, School of Basic Sciences, Indian Institute of Technology Mandi, Kamand, Himachal Pradesh, India; b Department of Molecular Biology and Biotechnology, Tezpur University, Tezpur, Assam, India; c Department of Computer Science & Engineering, Tezpur University, Tezpur, Assam, India; Princeton University

**Keywords:** *Ralstonia solanacearum*, ED pathway, ^13^C tracers, central metabolism, mass isotopomers, GC-MS, non-OxPPP

## Abstract

In Ralstonia solanacearum, a devastating phytopathogen whose metabolism is poorly understood, we observed that the Entner-Doudoroff (ED) pathway and nonoxidative pentose phosphate pathway (non-OxPPP) bypass glycolysis and OxPPP under glucose oxidation. Evidence derived from ^13^C stable isotope feeding and genome annotation-based comparative metabolic network analysis supported the observations. Comparative metabolic network analysis derived from the currently available 53 annotated R. solanacearum strains, including a recently reported strain (F1C1), representing the four phylotypes, confirmed the lack of key genes coding for phosphofructokinase (*pfk-1*) and phosphogluconate dehydrogenase (*gnd*) enzymes that are relevant for glycolysis and OxPPP, respectively. R. solanacearum F1C1 cells fed with [^13^C]glucose (99% [1-^13^C]glucose or 99% [1,2-^13^C]glucose or 40% [^13^C_6_]glucose) followed by gas chromatography-mass spectrometry (GC-MS)-based labeling analysis of fragments from amino acids, glycerol, and ribose provided clear evidence that rather than glycolysis and the OxPPP, the ED pathway and non-OxPPP are the main routes sustaining metabolism in R. solanacearum. The ^13^C incorporation in the mass ions of alanine (*m/z* 260 and *m/z* 232), valine (*m/z* 288 and *m/z* 260), glycine (*m/z* 218), serine (*m/z* 390 and *m/z* 362), histidine (*m/z* 440 and *m/z* 412), tyrosine (*m/z* 466 and *m/z* 438), phenylalanine (*m/z* 336 and *m/z* 308), glycerol (*m/z* 377), and ribose (*m/z* 160) mapped the pathways supporting the observations. The outcomes help better define the central carbon metabolic network of R. solanacearum that can be integrated with ^13^C metabolic flux analysis as well as flux balance analysis studies for defining the metabolic phenotypes.

**IMPORTANCE** Understanding the metabolic versatility of Ralstonia solanacearum is important, as it regulates the trade-off between virulence and metabolism (1, 2) in a wide range of plant hosts. Due to a lack of clear evidence until this work, several published research papers reported on the potential roles of glycolysis and the oxidative pentose phosphate pathway (OxPPP) in R. solanacearum (3, 4). This work provided evidence from ^13^C stable isotope feeding and genome annotation-based comparative metabolic network analysis that the Entner-Doudoroff pathway and non-OxPPP bypass glycolysis and OxPPP during the oxidation of glucose, a component of the host xylem pool that serves as a potential carbon source (5). The outcomes help better define the central carbon metabolic network of R. solanacearum that can be integrated with ^13^C metabolic flux analysis as well as flux balance analysis studies for defining the metabolic phenotypes. The study highlights the need to critically examine phytopathogens whose metabolism is poorly understood.

## Abstract (precis)

Understanding the metabolic versatility of Ralstonia solanacearum is important, as it regulates the trade-off between virulence and metabolism (1, 2) in a wide range of plant hosts. Due to a lack of clear evidence until this work, several published research papers reported on the potential roles of glycolysis and the oxidative pentose phosphate pathway (OxPPP) in R. solanacearum (3, 4). This work provided evidence from ^13^C stable isotope feeding and genome annotation-based comparative metabolic network analysis that the Entner-Doudoroff pathway and non-OxPPP bypass glycolysis and OxPPP during the oxidation of glucose, a component of the host xylem pool that serves as a potential carbon source (5). The outcomes help better define the central carbon metabolic network of R. solanacearum that can be integrated with ^13^C metabolic flux analysis as well as flux balance analysis studies for defining the metabolic phenotypes. The study highlights the need to critically examine phytopathogens whose metabolism is poorly understood.

## INTRODUCTION

Ralstonia solanacearum is one of the most destructive plant pathogens as it infects over 450 plant species (6–8), and its metabolism is poorly understood. It is a soilborne pathogen that enters plants through natural openings or wounds, colonizes, and blocks water conduction in the xylem, which leads to wilting and the death of plants (9). Recent studies showed that there is a resource allocation or trade-off between metabolism and virulence (1–2). Carbon metabolism not only plays a significant role in bacterial growth but is also involved in extracellular polysaccharide production and *hrp* gene expression, which are crucial for virulence and pathogenicity (10, 11). While the system features of virulence are widely studied, no detailed investigations on the central metabolism of R. solanacearum has been undertaken. Owing to its wide host range wherein the pathogen encounters various nutritional regimes, it is very important to understand the metabolic features of R. solanacearum. Metabolic versatility of the bacterium plays a crucial role in conferring growth as well as pathogenicity in its complex life cycle. This is supported by studies on the differential expression of genes in pathogenic and nonpathogenic strains of R. solanacearum wherein about 50% of the genes belong to carbohydrate and amino acid metabolism (9–14). Annotations based on genome analysis and stable isotope labeling can map the central metabolic pathways of R. solanacearum. Mapping of the oxidation of different carbon sources via the central metabolic pathways will shed light on how the cellular demands of NADPH, ATP, and other cofactors are met via metabolism, eventually defining the metabolic phenotypes.

The rich repository of available genomes of different R. solanacearum phylotypes or strains obtained via next-generation sequencing (NGS) can readily be annotated and comparatively analyzed using bioinformatics to decode the metabolic features. Currently, the genome data of 127 R. solanacearum strains are available in the database of the National Center for Biotechnology Information (NCBI), of which 47 are complete genome sequences, 13 are chromosome sequences, 32 are scaffolds, and 35 are contigs (as of October 2019 [https://www.ncbi.nlm.nih.gov/genome/genomes/490]). Also, 10 R. solanacearum annotated genomes are available in the KEGG (Kyoto Encyclopedia of Genes and Genomes) (15) and MicroScope/Genoscope databases (16). In this study, 53 complete genome sequences of R. solanacearum covering phylotypes I, II, III, and IV were selected for comparative pathway analysis. In addition, 10 other *Ralstonia* species strains were included for analysis. Studies have shown that the R. solanacearum genome is around 5 to 6 MB with a chromosome and a megaplasmid that houses various genes related to virulence, metabolism, regulatory, and secretory systems.

In this work, comparative genome analysis predicted the absence of some key pathway genes and enzymes (see Results section) whose validations of the pathway activities were further derived from ^13^C tracer-based mapping studies. Given the complexity of carbon transitions in the networks, often parallel ^13^C feeding experiments were needed to get a clear understanding of the pathways as well as to generate experimental evidence to confirm the missing genes, if any. For ^13^C-based retro-biosynthetic mapping of metabolic pathway activities, gas chromatography-mass spectroscopy (GC-MS)-based isotopomer analysis of different amino acid fragments derived from protein hydrolysates was needed (17). The experimental plan, selection of stable isotopes, and measurement of the isotope labeling in carbon atoms of the metabolites are crucial steps for pathway mapping using a ^13^C approach (18–19).

Since glucose is one of the preferred carbon sources available in the host xylem pool for R. solanacearum metabolism (2, 5), we adopted the methods of comparative genome and ^13^C mapping studies to validate and map the central metabolic pathways: glycolysis, the oxidative pentose phosphate pathway (OxPPP), the nonoxidative pentose phosphate pathway (non-OxPPP), and the Entner-Doudoroff (ED) pathway. We subjected R. solanacearum cells to labeling with [1-^13^C]glucose, [1,2-^13^C]glucose, and [^13^C_6_]glucose and investigated the label incorporation in the amino acids to gain comprehensive insight into the glucose oxidation pathways of central metabolism. The study generated comprehensive evidence to conclude that the Entner-Doudoroff pathway and non-OxPPP bypass glycolysis and OxPPP in Ralstonia solanacearum.

## RESULTS

### Comparative pathway analysis of Ralstonia solanacearum strains highlighted the absence of key central metabolic pathway genes.

**FIG 1:**
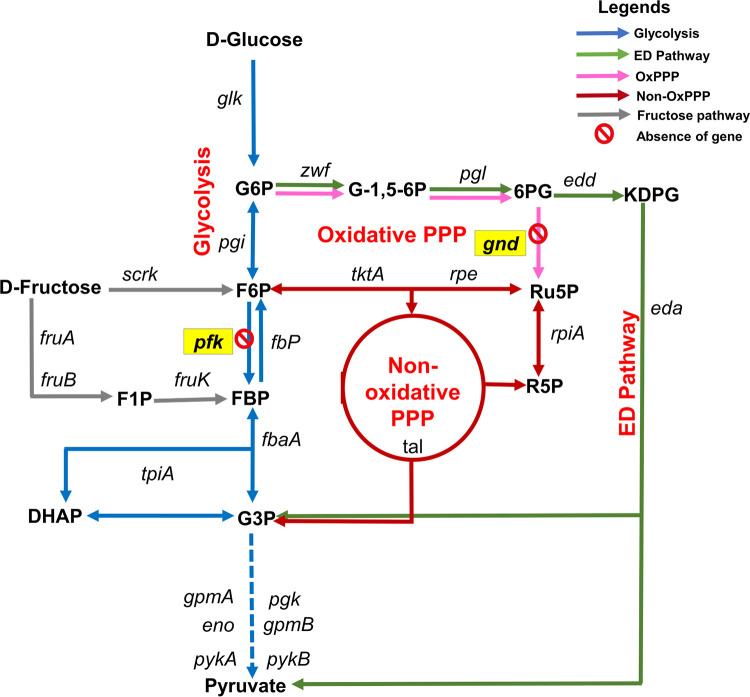
Glucose oxidation pathways in Ralstonia solanacearum strains highlight the absence of key pathway genes. Central metabolic pathways consisting of glycolysis, the Entner-Doudoroff (ED), and the pentose phosphate (PP) pathways among 63 strains of *Ralstonia* spp. were mapped for the annotation of relevant genes based on comparative genome analysis (see Table S1 in the supplemental material). All Ralstonia solanacearum strains (53) lacked glycolysis and the oxidative pentose phosphate pathway (OxPPP) genes *pfk* and *gnd* that code for phosphofructokinase and 6PG dehydrogenase, respectively. Among all the 63 strains of *Ralstonia* spp. studied, only R. pickettii (Table S1) showed the presence of *pfk*. The missing genes are highlighted in yellow boxes (*gnd* and *pfk*). Reactions between metabolites in the pathways, namely, glycolysis, ED pathway, OxPPP, non-OxPPP, and fructose metabolic pathway are indicated. Genes coding for the respective enzymes to catalyze the reactions and metabolites are presented in abbreviated form. The abbreviations used are the following: G6P, glucose-6-phosphate; F6P, fructose-6-phosphate; FBP, fructose 1,6-bisphosphate; G3P, glyceraldehyde 3-phosphate; DHAP, dihydroxyacetone phosphate; G-1,5-6P, glucono-1-5-lactone-6-phosphate; 6PG, phosphogluconate; KDPG, 2-keto-3-deoxy-6-phosphogluconate; Ru5P, ribulose 5-phosphate; R5P, ribose 5-phosphate; *glk*, glucokinase; *pgi*, glucose-6-phosphate isomerase; *pfk*, phosphofructokinase; *tpi*, triosephosphate isomerase; *pgk*, phosphoglycerate kinase; *gpm*, phosphoglycerate mutase; *eno*, enolase; *pyk*, pyruvate kinase; *zwf*, G6P dehydrogenase; *pgl*, phospho-gluconolactonoase; *edd*, phosphogluconate dehydratase; *gnd*, 6PG dehydrogenase; *eda*, 2-dehydro-3-deoxyphosphogluconate aldolase, *tkt*, transketolase; *rpe*, ribulose-phosphate 3-epimerase; *rpi*, ribose 5-phosphate isomerase; *tal*, transaldolase; *fbp*, fructose-1,6-bisphosphatase I; *fruK*, 1-phosphofructokinase; *fruA* and *fruB*, phosphotransferase system, enzyme I, *scrk*, fructokinase; *fba*A, fructose bisphosphate aldolase, class II.

Central metabolic pathways (glycolysis, Entner-Doudoroff [ED] pathway, and pentose phosphate pathway) of the selected organisms were reconstructed and visualized based on KEGG Orthology (KO) identifiers using a KEGG pathway reconstruction tool. The analysis confirmed the completeness of ED and non-OxPPP reactions (Fig. 1; see also Table S1 in the supplemental material). It was observed that all of the selected *Ralstonia* spp. (except R. pickettii) lacked the *pfk* gene that codes for an important regulatory enzyme, phosphofructokinase-1 (EC 2.7.1.11, KO0850), indicating the possible absence of glucose oxidation by the glycolytic pathway. Also, the *gnd* gene coding for phosphogluconate dehydrogenase enzymes (EC 1.1.1.44 and EC 1.1.1.343, KO0033) is absent in all *Ralstonia* spp. studied, indicating an incomplete OxPPP (Table S1). Experimental evidence in relation to the absence of these pathways in R. solanacearum was generated by parallel feeding of multiple [^13^C]glucose substrates and tracking the label redistribution.

10.1128/mSystems.00091-20.4 TABLE S1 Comparative pathway analysis of 53 Ralstonia solanacearum strains and 10 strains from different *Ralstonia* spp. along with E. coli based on presence and absence of key pathway genes for glucose oxidation namely, glycolysis, ED pathway, and pentose phosphate pathway. Black boxes represent the absence of genes. All *Ralstonia* species strains studied lack the *pfk-1* gene (supports glycolysis) except for R. pickettii, while the *gnd* gene (supports OxPPP) is absent in all of the *Ralstonia* spp. E. coli, which has all of the glucose oxidation pathways (glycolysis, ED pathway, and pentose phosphate pathway) intact, was used as a control. Download Table S1, PDF file, 0.3 MB. Copyright © 2020 Jyoti et al. 2020 Jyoti et al. This content is distributed under the terms of the Creative Commons Attribution 4.0 International license.

### Amino acid fragments reporting on central metabolic pathways identified.

**FIG 2:**
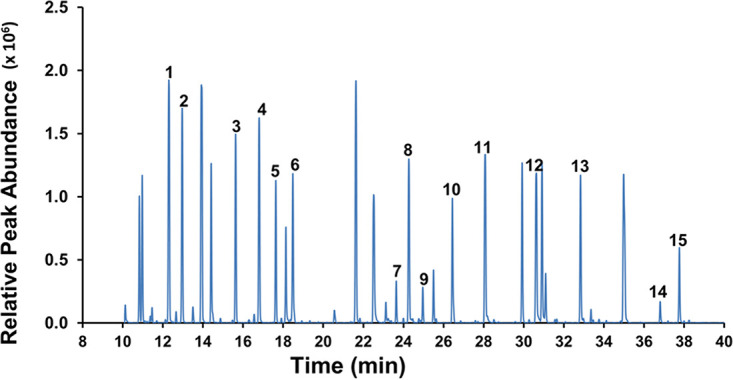
Total ion chromatogram of TBDMS-derivatized protein hydrolysate of R. solanacearum obtained by GC-MS. The peaks corresponding to the detected amino acids, with their respective elution times (in minutes), are the following, in numerical order from 1 to 15: alanine (12.285), glycine (12.958), valine (15.622), leucine (16.793), isoleucine (17.629), proline (18.476), methionine (23.632), serine (24.262), threonine (24.955), phenylalanine (26.436), aspartic acid (28.068), glutamic acid (30.612), lysine (32.826), histidine (36.793), and tyrosine (37.752). The retention time, derivative, specific ions, and carbon backbone of each amino acid fragment detected are presented in Table S2 in the supplemental material.

The tertiary butyl dimethyl silane (TBDMS)-derivatized protein hydrolysate of cells fed with [^12^C]glucose, [1-^13^C]glucose, [1,2-^13^C]glucose, and 40% [^13^C_6_]glucose were subjected to GC-MS for accurate analysis of mass isotopomer distribution in amino acids. The total ion chromatogram of protein hydrolysate resulted in 15 amino acids (Fig. 2). Each TBDMS-derivatized amino acid due to ionization formed a different fragment in the mass spectroscopy, such as [M-0]^+^, [M-15]^+^, [M-57]^+^, [M-85]^+^, [M-159]^+^, and [M-R]^+^ (R denotes the side chain of an amino acid often resulting in fragment [f302]^+^). The 15 amino acids with their respective elution times (minutes) and the reliable mass ions (*m/z*) of fragments [M-85] and [M-57] along with the carbon numbers are presented in Table S2.

10.1128/mSystems.00091-20.5 TABLE S2 Amino acid fragments of R. solanacearum. All of the detected 15 amino acids with their respective peak numbers, elution time (Fig. 1), derivative, and specific ions (*m*/*z*) obtained from GC-MS are tabulated. Download Table S2, PDF file, 0.07 MB. Copyright © 2020 Jyoti et al. 2020 Jyoti et al. This content is distributed under the terms of the Creative Commons Attribution 4.0 International license.

We observed that the [M-57] fragment ion contains all the backbone carbons of an amino acid, while the first carbon is missing in the [M-85] fragment. The fragments that are valid for further analysis were derived based on average ^13^C analysis (Table S3). The average ^13^C abundances (in percent) of each amino acid fragment as well as relative mass isotopomer distributions (MIDs) used for pathway mapping are presented in Tables S3 and S4, respectively. The ^13^C feeding studies have shown substantial isotope label incorporation in the amino acids of R. solanacearum. In cells fed with 40% [^13^C_6_]glucose, most of the amino acids except glycine resulted in average ^13^C enrichment to the extent of 35%. In the case of glycine, the average ^13^C labeling was ∼20%, highlighting the potential dilution from either external CO_2_ or methylenetetrahydrofolate due to a glycine synthase reaction, wherein glycine is cleaved to CO_2_, NH^4+^, and a methylene group (-CH_2_-), which is accepted by tetrahydrofolate (THF) in a reversible reaction (20). The mass isotopomers of amino acids derived from positional [1-^13^C]glucose- and [1,2-^13^C]glucose-fed R. solanacearum cells under the same conditions as that of [^13^C_6_]glucose supported comprehensive analysis.

10.1128/mSystems.00091-20.6 TABLE S3 Average 13C abundance (in percentages) of Ralstonia solanacearum F1C1 strain (valid, [M-57] and [M-85] amino acid fragment ions and standard deviation from 4 replicates) subjected to minimal medium supplemented with [^12^C_6_]glucose, [^1-13^C]glucose, [^1,2-13^C]glucose, and [^13^C_6_]glucose. Download Table S3, PDF file, 0.1 MB. Copyright © 2020 Jyoti et al. 2020 Jyoti et al. This content is distributed under the terms of the Creative Commons Attribution 4.0 International license.

10.1128/mSystems.00091-20.7 TABLE S4 The mass isotopomer distributions (MIDs) of amino acid fragments (valid, [M-57] and/or [M-85] and standard deviation from 4 replicates) of Ralstonia solanacearum F1C1 subjected to minimal medium supplemented with [^12^C_6_]glucose, [^1-13^C]glucose, [^1,2-13^C]glucose, and [^13^C_6_]glucose. Download Table S4, PDF file, 0.1 MB. Copyright © 2020 Jyoti et al. 2020 Jyoti et al. This content is distributed under the terms of the Creative Commons Attribution 4.0 International license.

### ED pathway and non-OxPPP bypass glycolysis and OxPPP.

**FIG 3:**
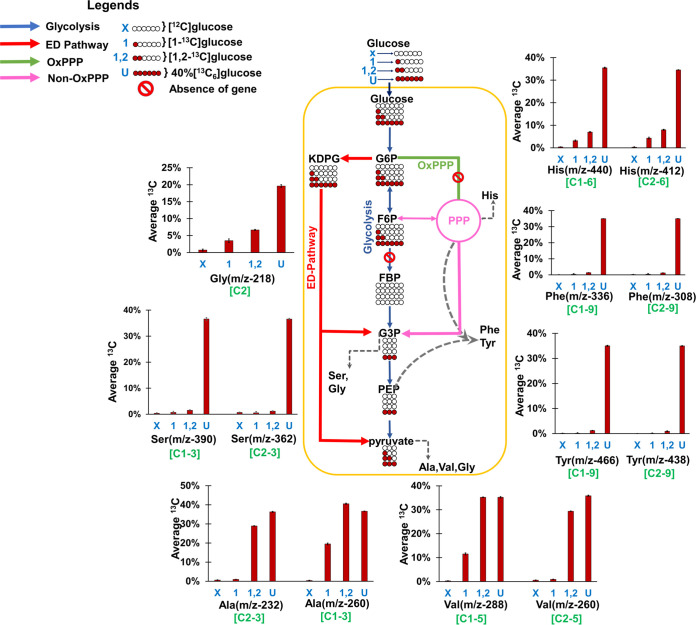
Average ^13^C label incorporation in the amino acids of R. solanacearum F1C1. Cells were fed with [^12^C]glucose (X), [1-^13^C]glucose (1), [1,2-^13^C]glucose (1,2), and 40% [^13^C_6_]glucose (U). The average percentages of ^13^C in amino acids ([M-57] and/or [M-85] fragments from GC-MS) highlight the extent of label incorporation and further retro-biosynthetically report on each metabolite precursor as well as pathway activities. In the case of [^12^C]glucose, the average percentages of ^13^C in all fragments were at natural abundance (∼1.13%). The average ^13^C values in alanine, valine, serine, and glycine from all parallel feeding experiments confirm the activity of the ED pathway, which bypasses glycolysis (see the Results section for a detailed explanation). The circles below the metabolite labels correspond to the carbon backbone, with each position either labeled (red filled) or unlabeled (white) corresponding to a feeding experiment, as indicated in the legend. The reactions, namely, glycolysis, ED (Entner-Doudoroff) pathway, oxidative pentose phosphate pathway (OxPPP), and the reductive pentose phosphate pathway are indicated in presented in blue, red, green, and pink, respectively; amino acids are indicated in dotted gray lines. The abbreviations used for the metabolites are as follows: G6P, glucose-6-phosphate; F6P, fructose-6-phosphate; FBP, fructose 1,6 bisphosphate; G3P, glyceraldehyde 3-phosphate; PEP, phosphoenolpyruvate; KDPG, 2-keto-3-deoxy-6-phosphogluconate; Ala, alanine; Val, valine; Ser, serine; Gly, glycine; Tyr, tyrosine; Phe, phenylalanine; His, histidine.

**FIG 4:**
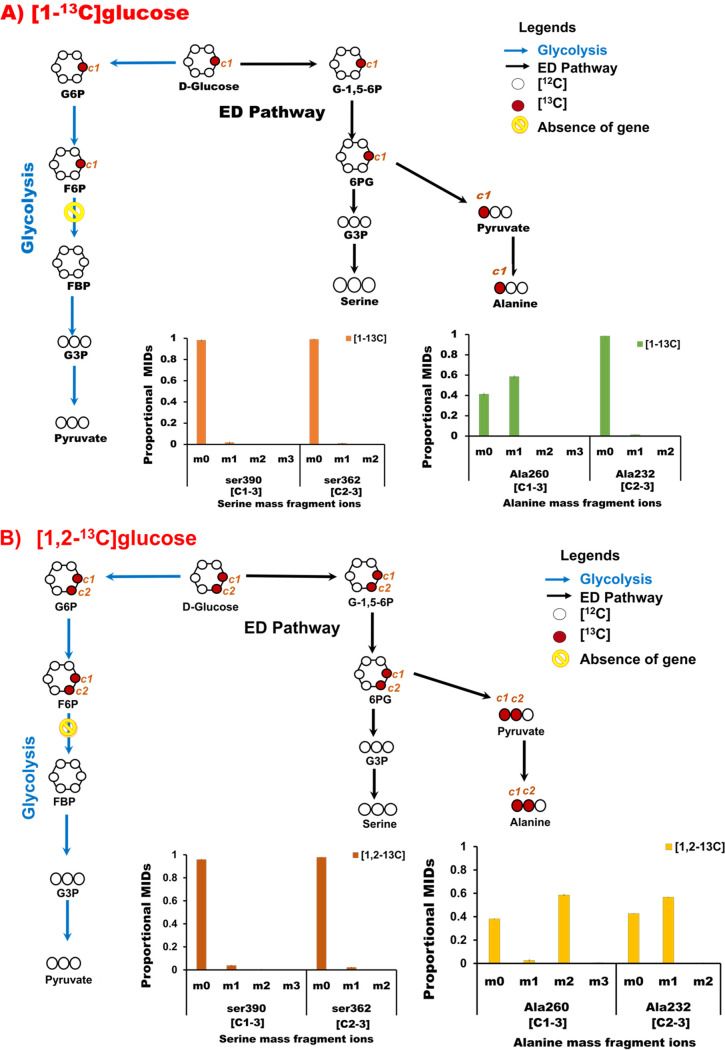
Tracking of ^13^C label in serine and alanine through glycolysis and the ED pathway in R. solanacearum F1C1. The label enrichment in the pathway intermediates interpreted from the mass isotopomers of serine and alanine derived from cells fed in parallel with [1-^13^C]glucose (A) and [1,2-^13^C]glucose (B) is presented. The mass isotopomer distributions in serine *m/z* 390 (C-1 to C-3) and *m/z* 362 (C-2 and C-3) and alanine *m/z* 260 (C-1 to C-3) and *m/z* 232 (C-2 and C-3) show that the ED pathway is the predominant route of glucose oxidation with no appreciable activity of glycolysis ([1-^13^C]glucose oxidation via glycolysis), which would result in labeled serine, whereas the ED pathway would result in unlabeled serine, which is the case. Cells fed with [1,2-^13^C]glucose would also result in a similar labeling pattern. Abbreviations: G-1,5-6P, glucono-1-5-lactone-6-phosphate; 6PG, phosphogluconate; G6P, glucose 6-phosphate; F6P, fructose 6-phosphate; G3P, glyceraldehyde 3-phosphate; MID, mass isotopomer distribution.

**FIG 5:**
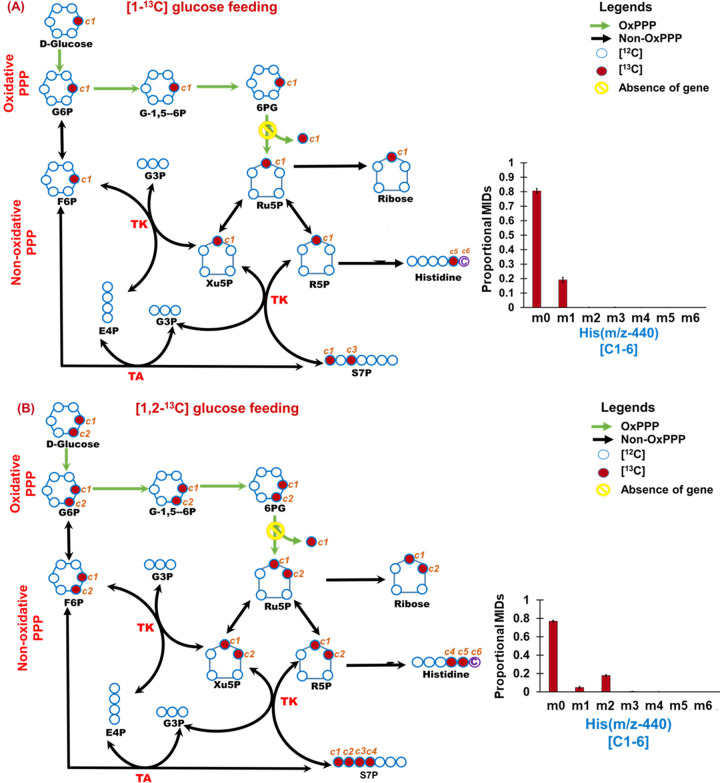
Movement of ^13^C label through OxPPP and non-OxPPP in R. solanacearum F1C1. The carbon transition and label incorporation in the pathway intermediates were interpreted from the mass isotopomers of histidine derived from cells fed in parallel with [1-^13^C]glucose (A) and [1,2-^13^C]glucose (B). The mass isotopomer distribution in histidine *m/z* 440 (C-1 to C-6) shows that non-OxPPP is the predominant route of glucose oxidation with no appreciable activity of OxPPP. [1-^13^C]glucose oxidation via OxPPP would result in unlabeled histidine, whereas non-OxPPP would result in one carbon-labeled (m+1) histidine, which is the case in these cells. Similarly, it is observed that cells fed with [1,2-^13^C]glucose exhibited higher levels of m+2 in histidine, which is feasible via the non-OxPPP (see the Results section for details). Abbreviations: G-1,5-6P, glucono-1-5-lactone-6-phosphate; 6PG, phosphogluconate; Ru5P, ribulose 5-phosphate; R5P, ribose 5-phosphate; Xu5P, xylulose 5-phosphate; F6P, fructose 6-phosphate; G3P, glyceraldehyde 3-phosphate; E4P, erythrose 4-phosphate; S7P, sedoheptulose 7-phosphate; PRPP, phosphor ribose diphosphate; MID, mass isotopomer distribution.

Average ^13^C incorporation in amino acid fragments retro-biosynthetically provided information on central metabolite precursors, thereby highlighting the glucose oxidation pathway activities in R. solanacearum mainly via the ED pathway and non-OxPPP (Fig. 3 to 5; Table S3 and S4). Mainly the label incorporation in the [M-57] and/or [M-85] fragment of alanine *m/z* 260 and *m/z* 232, valine *m/z* 288 and *m/z* 260, glycine *m/z* 218, serine *m/z* 390 and *m/z* 362, histidine *m/z* 440 and *m/z* 412, tyrosine *m/z* 466 and *m/z* 438, phenylalanine *m/z* 336 and *m/z* 308, glycerol *m/z* 377, and ribose *m/z* 160 provided the relevant evidence.

When cells were fed with [1-^13^C]glucose, it was observed that alanine (19.5% average ^13^C), valine (11.5%), glycine (3.5%), and histidine (4%) were labeled while no ^13^C enrichment was observed in serine, phenylalanine, and tyrosine. The presence of unlabeled serine, phenylalanine, and tyrosine indicates that carbon movement is not via glycolysis. The higher labeling in alanine *m/z* 260 ([M-57] fragment ion containing C-1 to C-3 in the carbon backbone having 19.5% average ^13^C) than in the alanine *m/z* 232 ([M-85] fragment ion with C-2 and C-3 in the carbon backbone with no significant label) indicates that the C-1 position of alanine (or its precursor pyruvate) is labeled, which is mainly possible via the ED pathway (Fig. 3 and 4; Table S3). The ED pathway activity as well as the lack of glycolytic activity was further supported by [1,2-^13^C]glucose feeding data of alanine *m/z* 260 (C-1 to C-3, 40% average ^13^C), alanine *m/z* 232 (C-2 and C-3, 29% average ^13^C), serine *m/z* 390 (C-1 to C-3, 2% average ^13^C), and serine *m/z* 362 (C-2 and C-3, 1% average ^13^C) (Fig. 3; Table S3).

If glycolysis were active, we would have observed ^13^C incorporation in serine (or phosphoglutamate [PGA] or glyceraldehyde 3-phosphate [G3P]) and the C-3 position of alanine (or pyruvate) from [1-^13^C]glucose and [1,2-^13^C]glucose feeding experiments, which was not the case (Fig. 4A and B). We also checked the ^13^C enrichment in glycerol (*m/z* 377, mapped to glyceraldehyde 3-phosphate, the nearest measurable product to serine) from [1-^13^C]glucose and [1,2-^13^C]glucose feeding experiments and found no significant labeling (Fig. S3), further supporting inactive glycolysis. This clearly provides evidence that ED pathway bypasses glycolysis wherein the labeled C-1 of glucose ends in pyruvate and subsequently in alanine and valine. The analysis clearly supports the notion that the lack of the *pfk* gene in R. solanacearum is responsible for the inactivity of glycolysis, with the ED pathway as one of the preferred glucose oxidation routes.

We also observed that non-OxPPP bypasses OxPPP, as evidenced from both [1-^13^C]glucose and [1,2-^13^C]glucose feeding experiments (Fig. 5). The activities of PPP were tracked retro-biosynthetically from histidine (*m/z* 440 [C-1 to C-6] whose carbons are derived from ribose-5P and formate) and ribose (*m/z* 160 [C-1 and C-2]). Mass isotopomer distribution of histidine (Fig. 5; Table S4) represented by mass isotopomers (like m+0, m+1 to m+6) and relative proportions of mass ion abundances of ribose (Fig. S3) have shed light on the PPP. From the cells fed with [1-^13^C]glucose, the labeling was observed in the mass isotopomer m+1 in histidine (*m/z* 440), which is possible by the activity of the reductive pentose phosphate pathway (i.e., non-OxPPP). If OxPPP is active, it is expected that there would be loss of C-1 from [1-^13^C]glucose and that we would obtain unlabeled ribose and histidine, which is not the case. However, if both non-OxPPP and OxPPP are active, we can still expect label incorporation in the m+1 of histidine (*m/z* 440), which suggests that [1-^13^C]glucose feeding alone cannot confirm the inactivity of OxPPP. To further confirm the relative contributions of non-OxPPP and OxPPP, the cells were also fed with [1,2-^13^C]glucose. The label incorporation showed higher levels of m+2 than m+1, which confirms that a ^13^C label in ribose and histidine is mainly contributed via non-OxPPP (Fig. 5; Fig. S3). The data support the idea that non-OxPPP, rather than OxPPP, is predominantly active, possibly due to the lack of the *gnd* gene (or appreciable OxPPP activity) in R. solanacearum strains, as confirmed via comparative genome analysis (Fig. 1).

## DISCUSSION

Ralstonia solanacearum is a devastating phytopathogen, and it is very important to understand its complete central metabolic network, mainly the elucidation of carbon metabolism. With the aim to investigate the intactness of all of the central carbon pathways in R. solanacearum, we undertook pathway comparisons of different strains (representing all four phylotypes, I, II, III, and IV) derived from 53 genomes available in NCBI, MicroScope/Genoscope, and KEGG, against the recently reported F1C1 strain (21), which we sequenced and annotated. In addition, 10 strains covering *Ralstonia* species other than Ralstonia solanacearum were considered for comparative pathway analysis (see Table S1 in the supplemental material).

It was observed that most of the *Ralstonia* spp. have some genes missing in the glycolysis pathway and the OxPPP, which raises curiosity about how this genus sustains its metabolism. All of the R. solanacearum strains lacked the *pfk* gene that encodes an important regulatory enzyme, phosphofructokinase-1 (EC 2.7.1.11, KO0850), which indicates possible bypass of the glycolytic pathway by alternative routes. Also, the *gnd* gene coding for phosphogluconate dehydrogenase enzymes (EC 1.1.1.44 and EC 1.1.1.343, KO0033) is absent in all R. solanacearum strains, indicating an incomplete OxPPP (Fig. 1). R. solanacearum was earlier placed in the genus *Pseudomonas* (7), and it was reported that most *Pseudomonas* species lack glycolysis (22). Cupriavidus necator (previously classified as Ralstonia eutropha) lacks the enzyme PFK-1 and uses the ED pathway as an alternative to glycolysis (23). Although the possibility of alternative pathways seems evident from genome annotations, experimental validation of *in vivo* activities providing evidence of the absence of glycolysis and OxPPP in R. solanacearum is warranted. We selected a ^13^C isotope labeling approach to get deeper insights into the central carbon metabolic pathways and to validate the bypassing of pathways as well as confirming the lack or extent of glycolytic and OxPPP activities in R. solanacearum F1C1. Parallel tracer feeding experiments using ^13^C-labeled glucose (99% [1-^13^C]glucose or 99% [1,2-^13^C]glucose or 40% [^13^C_6_]glucose) were undertaken for comprehensive insights into pathway activities. In terms of the approaches used, ^13^C incorporation in the carbons of amino acids retro-biosynthetically provides information on ^13^C incorporation in precursors and other central metabolites. Parallel ^13^C labeling experiments (using [1-^13^C]glucose, [1,2-^13^C]glucose, and [^13^C_6_]glucose) were conducted, which are standard practice and are robust.

Cells fed with [1-^13^C]glucose have ^13^C enrichment in pyruvate, but there is no ^13^C incorporation in serine and glycerol (Fig. 3 and 4; Tables S3 and S4 and Fig. S3), indicating the lack of glycolysis and an active ED pathway, which is further supported by labeling in the C-1 of alanine (19, 24). The equal distribution of average ^13^C levels (in percent) in serine and alanine from [^13^C_6_]glucose feeding is another indication of mainly ED pathway activity (Fig. 3; Tables S3 and S4). Higher ^13^C enrichment in the [M-57] fragment of alanine (*m/z* 260) than in the [M-85] fragment (alanine *m/z* 232) when [1-^13^C]glucose was used is confirmation of the active ED pathway (25). Similar results were reported in Dinoroseobacter shibae fed with [1-^13^C]glucose (25).

The activity of the oxidative pentose phosphate pathway is questionable in R. solanacearum as comparative genome analysis highlighted the absence of the key gene for 6-phosphogluconate (6PG) dehydrogenase (*gnd*; EC 1.1.1.44) (Fig. 1). To validate this, we undertook parallel [1-^13^C]glucose and [1,2-^13^C]glucose feeding and measured the ^13^C incorporation in histidine and ribose that can retro-biosynthetically report on the PPP activities. The carbon precursors for histidine biosynthesis are 5-phospho-ribosyl-a-pyrophosphate (PRPP) and the formyl group of *N^1^*^0^-formyl-tetrahydrofolate (THF) (24). In OxPPP, the first (labeled) carbon in [1-^13^C]glucose is generally released as ^13^CO_2_ during the conversion of 6-phosphogluconate into d-ribulose-5-phosphate by *gnd*. In R. solanacearum, if OxPPP is predominantly active, then it is expected not to incorporate any ^13^C in histidine. However, ^13^C incorporation in both histidine and ribose were observed, which explains that the predominant activity is due to the nonoxidative or reductive PPP (non-OxPPP) (Fig. 5, Table S4, and Fig. S3). To further confirm that ^13^C label is from the non-OxPPP, bacterial cells were also fed with [1,2-^13^C]glucose. It was observed that the mass isotopomer abundances in histidine (m + 2) and relative proportions of mass representing ribose (m + 2) were higher than m + 1 (Fig. 5 and Fig. S3), which confirms that non-OxPPP, rather than the OxPPP, is predominantly active, possibly due to the lack of the *gnd* gene in R. solanacearum.

In addition, on close examination of the publicly available transcriptome data of R. solanacearum strains GMI1000 and UW551 derived from *in planta* and rich-medium growth (4), it was found that *pfk-1* and *gnd* gene expression levels were not reported (while several other genes such as those of the ED pathway were expressed), potentially owing to their absence. Similarly, the transcriptome analysis by Puigvert et al. has no reports of *gnd* and *pfk-1* genes in homologous gene analysis of R. solanacearum strains whereas ED pathway genes were reported (26). These observations further supported our findings that the *gnd* and *pfk-1* genes are inactive or altogether absent in R. solanacearum and that mainly glucose oxidation bypasses glycolysis and OxPPP in favor of the ED pathway and non-OxPPP.

Overall, this work, which is supported by comparative pathway analysis from genomes and parallel ^13^C labeling analysis, suggests that the ED pathway and non-OxPPP are the main metabolic routes used by R. solanacearum for glucose oxidation. The ED pathway plays significant roles in the activation of virulence factors and inhibition of biofilm formation (27). Biofilm aids in survival of the pathogen and helps the pathogen enter the host, but inside the host, bacterial cells disperse and form individual colonies for infection. Although the ED pathway produced one ATP (less than glycolysis), there are several microbes that prefer the ED pathway over glycolysis, probably due to some evolutionary advantage. To explore the reason, Flamholz and coworkers (28) studied the energy yield and protein cost in the glycolytic and ED pathways in prokaryotes. They hypothesized that the enzymatic synthesis cost also plays a key role in the choice of the pathway used for glucose catabolism. The use of the ED pathway results in a lower protein synthesis cost than glycolysis to catabolize the same amount of glucose (28). Similarly, the lack of OxPPP activity would starve the cells of NADPH, which may be compensated by alternative pathways producing NADPH or reducing the cellular needs of this valuable resource. It remains to be seen how R. solanacearum adjusts to its metabolic demands under the varied nutritional niches it encounters. The findings reported here will be of immense relevance in defining the metabolic phenotypes of R. solanacearum. This is the first comprehensive ^13^C labeling study in R. solanacearum with a focus on deciphering its glucose oxidation and on mapping the central carbon metabolic pathways in R. solanacearum.

## MATERIALS AND METHODS

### Chemicals.

The labeling substrates, [1-^13^C]glucose (99 atom%), [1,2-^13^C]glucose (99 atom%), and [^13^C_6_]glucose (99 atom%), were purchased from Sigma-Aldrich. Derivatization agents TBDMS [*N*-methyl-*N*-(*t*-butyldimethylsilyl) trifluoroacetamide plus 1% *t*-butyl-dimethylchlorosilane] and MSTFA [*N*-methyl-*N*-(trimethylsilyl) trifluroacetamide] were also purchased from Sigma-Aldrich. Medium components and other reagents were purchased from Himedia and Sigma-Aldrich.

### Software.

KEGG Mapper, Agilent ChemStation, MassHunter, IsoCorr, MetAlign, and AMDIS (National Institute of Standards and Technology) software programs were used. Online resources were also used.

### Analytical equipment.

The GC-MS instrument was from Agilent, and an ELx808 microplate reader was from Bio-Tek Instruments, Inc.

### Experimental procedures.

**(i) Comparative metabolic pathway analysis.** For comparative analysis of metabolic pathways, genome sequences of 53 strains of Ralstonia solanacearum including F1C1 (unpublished data), which belong to different phylotypes (representing all four phylotypes, I, II, III, and IV) were selected (see Table S1 in the supplemental material). Six other *Ralstonia* spp. (covering 10 strains) were selected: R. syzygii strain R24, R. insidiosa strain FC1138, R. eutropha strain JMP134 (now renamed Cupriavidus necator strain H16), banana blood disease strain R229, R. pseudosolanacearum strain RS 476, R. mannitolilytica strain SN82F48, and R. pickettii strains 12D, DTP0602, and 12J. Escherichia coli strain DH10B was used as a positive control. Central metabolic pathways were compared based on KEGG pathway mapping (15). For the organisms not present in the KEGG database, the multi-FASTA amino acid sequences were downloaded from Genoscope/MicroScope (16) or NCBI (29). Then, KO identifiers from these amino acid FASTA files were generated using BlastKOALA (BLAST-based KO annotation and KEGG mapping) (30). Comparative pathway analysis and visualization of selected *Ralstonia* spp. along with E. coli were done using the KEGG pathway reconstruction tool (30). Bacto peptone, 10; Casamino Acids, 1; yeast extract.

**(ii) Cell growth and maintenance.** R. solanacearum F1C1 culture was maintained in BG medium (bactoagar 15 g/liter, glucose 5 g/liter) in 50-ml falcon tubes at 28°C on an orbital shaker running at 180 rpm (31, 32). For pathway mapping experiments, cells were grown in BG medium for 24 h until the optical density at 600 nm (OD_600_) reached 1. Cells were pelleted down by centrifugation at 5,000 rpm for 5 min. Cells were transferred to minimal medium and grown for 24 h to adapt in minimal medium. Cells were further harvested, washed with sterile water, and redistributed in minimal medium supplemented with labeled glucose (0.5%, wt/vol) with four replicates. The composition of minimal medium (14) is as follows (in grams/liter): FeSO_4_-7H_2_O, 1.25 × 10^−4^; (NH_4_)_2_SO_4_, 0.5; MgSO_4_-7H_2_O, 0.05; KH_2_PO_4_, 3.4. The pH was adjusted to 7.0 with 1 M KOH. Cells were grown in minimal medium with different combinations of labeled isotopes ([1-^13^C]glucose, [1,2-^13^C]glucose, 40% [^13^C_6_]glucose, and [^12^C]glucose, with four technical replicates of each condition) (Fig. S1). Cells were harvested at 18 h during mid-exponential phase that represents a pseudo-steady-state condition (Fig. S2).

10.1128/mSystems.00091-20.1 FIG S1 Experimental workflow adopted for central metabolic pathway mapping by isotopic tracer feeding and GC-MS data analysis toward average ^13^C label incorporation in proteinogenic amino acids. R. solanacearum cells were grown overnight in BG medium until the optical density (OD_600_) reached 1. Cells were harvested by centrifugation at 5,000 rpm and then transferred to minimal medium to adapt for 24 h; cells were again harvested and redistributed in minimal medium with isotopic tracers, i.e., 40% [^13^C_6_]glucose, [1-^13^C_6_]glucose, and [1,2-^13^C_6_]glucose, along with ^12^C substrates as a control (four replicates each). Cells were harvested after 18 h of inoculation and were acid hydrolyzed using 6 M HCl. Protein hydrolysates were derivatied by TBDMS (tertiary butyl dimethyl silane) derivatization for GC-MS analysis. The total ion chromatograms (TIC) of all amino acids were corrected for natural isotope correction and validation was based on average ^13^C content in unlabeled fragments. Validated amino acid fragments were then used to map the central metabolic pathways. Download FIG S1, TIF file, 1.1 MB. Copyright © 2020 Jyoti et al. 2020 Jyoti et al. This content is distributed under the terms of the Creative Commons Attribution 4.0 International license.

10.1128/mSystems.00091-20.2 FIG S2 The growth curve of Ralstonia solanacearum cells in minimal medium containing 0.5% (wt/vol) glucose as a carbon source. For metabolic pathway mapping, cells were harvested at 18 h during mid-exponential phase, which represents the pseudo-steady-state condition. Download FIG S2, TIF file, 0.7 MB. Copyright © 2020 Jyoti et al. 2020 Jyoti et al. This content is distributed under the terms of the Creative Commons Attribution 4.0 International license.

10.1128/mSystems.00091-20.3 FIG S3 The relative proportion of mass ions from fragments of ribose (*m*/*z* 160) (a and b) and glycerol (*m*/*z* 377) (c and d) of Ralstonia solanacearum F1C1 subjected to minimal medium supplemented with either [^12^C_6_]glucose, [1-^13^C]glucose, or [1,2-^13^C]glucose. The mean relative abundance of mass ions and corresponding standard deviations from 4 replicates are presented. The significant difference in relative proportions were analyzed by Student’s t test as follows: in ribose, *, *P* < 0.5; **, *P* < 0.01; ***, *P* < 0.001; and ****, *P* < 0.0001; in glycerol, *, *P* < 1; **, *P* < 0.5; ***, *P* < 0.001; and ****, *P* < 0.0001. The mass fragments are not corrected for ^13^C natural abundances. Download FIG S3, TIF file, 1.2 MB. Copyright © 2020 Jyoti et al. 2020 Jyoti et al. This content is distributed under the terms of the Creative Commons Attribution 4.0 International license.

**(iii) Acid hydrolysis and derivatization of amino acids.** Cell pellets (2 mg each) were acid hydrolyzed by suspending them in 500 μl of 6 M HCl for 18 h at 100°C to release the amino acids (33). The acid hydrolysate (50 μl) was then dried in a speed vacuum (Thermo Scientific, Waltham, MA) to ensure complete removal of water. The amino acids extracts were derivatized by TBDMS (34). To obtain the TBDMS derivatives, the vacuum-dried acid hydrolysates of amino acids were first dissolved in 30 μl of pyridine (Sigma-Aldrich) and incubated at 37°C, with shaking at 900 rpm, for 30 min. Then 50 μl of MtBSTFA plus 1% *t*-BDMCS [*N*-methyl-*N*-(*t*-butyldimethylsilyl) trifluoroacetamide plus 1% *t*-butyl-dimethylchlorosilane; Regis Technologies, Inc.) was added, and samples were incubated at 60°C with shaking at 900 rpm for 30 min on a thermomixer. The derivatized samples were centrifuged for 10 min at 13,000 rpm to pellet down any insoluble material, and the supernatant was transferred to glass vials for GC-MS and sealed with a septum cap.

**(iv) Sample preparation for ribose labeling.** The ^13^C enrichment in ribose was measured as described by Long and Antoniewicz (35). The dry cells (2 mg) were acid hydrolyzed with 50 μl of 6 N HCl for 30 min and then diluted to 1 N by addition of 250 μl of distilled water and incubated for 1 h in a fume hood. The reaction was neutralized by addition of 40 μl of 5 N NaOH. The reaction mixture was centrifuged at 14,000 rpm for 10 min. Fifty microliters of supernatant was dried in a speed vacuum (Thermo Scientific). To obtain the trimethylsilyl (TMS) derivatives, the vacuum-dried extracts were dissolved in 35 μl of freshly prepared hydrochloride (MeOX; Sigma-Aldrich) in pyridine (20 mg/ml) and incubated on a thermoshaker at 37°C at 900 rpm for 2 h. After that, 49 μl of MSTFA was added, and the samples were incubated at 37°C and 900 rpm for 30 min on a thermomixer (36). The derivatized samples were centrifuged for 10 min at 13,000 rpm, and the supernatant was transferred to glass vials for GC-MS.

**(v) Gas chromatography-mass spectrometry.** The GC-MS measurements were performed on an Agilent 7890B GC with electron impact ionization (70 eV), equipped with an Agilent HP 5-ms Ultra Inert column (Agilent 19091S-433UI, 30 m by 250 μm by 0.25 μm), at the facility in the BioX Center at the Indian Institute of Technology (IIT) Mandi. In GC-MS, 1 μl of sample volume was taken for injection. For TBDMS-derivatized amino acid hydrolysate, the initial oven temperature was constant at 120°C for 5 min and then ramped at 4°C/min to 270°C, held for 3 min, ramped at 20°C/min to 320°C, and held for 1 min. The carrier gas (helium) flow was maintained at 1.3 ml min^−1^. The spectra were recorded with a scanning range of 30 to 600 mz-1 for a total run time of 49 min. For MeOX-TMS derivatized samples, oven temperature was constant at 50°C for 5 min and then ramped at 10°C/min to 200°C and held for 10 min. Temperature was again ramped at 5°C/min to 300°C and held for 10 min. Afterwards the temperature was decreased to 70°C by 100°C/min. The carrier gas (helium) flow was 0.6 ml min^−1^. MassHunter (Agilent Technologies, USA) was used to control the data acquisition parameters (both GC separation and mass spectrometry) during all sample runs.

**(vi) Metabolite identification and mass isotopomer data handling.** The raw GC-MS spectra need to be baseline corrected at first for accurate assessment of mass isotopomer distributions in metabolites. The raw files from GC-MS were baseline corrected using MetAlign software (37) with its default parameters. Metabolite identification was done according to the National Institute of Standards and Technology (NIST, Gaithersburg, MD). The intensity of the mass ions of each amino acid fragment (38) was obtained by using Agilent ChemStation software. The MID of each fragment ion obtained from averaged mass spectra was corrected for the presence of naturally occurring heavy isotopes attached to the carbon backbone of the derivative using the mass correction software IsoCor (39). The corrected MID values were used to calculate the average ^13^C abundance in each fragment (34).

### Statistical analysis.

Four replicates were used in all of the analyses, and data are presented as the means ± standard deviations (SD) for each experiment. The statistical significance was determined using Student's *t* test.
