# Supplementary material for: The Entner-Doudoroff and Nonoxidative Pentose Phosphate Pathways Bypass Glycolysis and the Oxidative Pentose Phosphate Pathway in Ralstonia solanacearum
Source: mSystems. 2020 Mar 10;5(2):e00091-20. doi: 10.1128/mSystems.00091-20 (PMC7065512; doi:10.1128/mSystems.00091-20)
Supplement: TABLE S1 [file mSystems.00091-20-st001.pdf]

Supplementary Table S1. Comparative pathway analysis of 53 *Ralstonia solanacearum* strains and 10 strains from different *Ralstonia spp.* along with *E. coli* based on presence and absence of key pathway genes for glucose oxidation namely, glycolysis, ED-pathway and Pentose Phosphate Pathway. Black colored boxes represent the absence of genes. All *Ralstonia spp.* strains studied have absence of *pfk-1* gene (support glycolysis) except for *R. picketti* while *gnd gene* (support OxPPP) is absent in all the *Ralstonia spp. E. coli* was used as control which has all the glucose oxidation *pathways (*glycolysis, ED-pathway and Pentose Phosphate Pathway) intact.

		**Glycolysis Pathway genes**												**ED Pathway genes**				**OxPPP**			**Non-OxPPP**				**Ref**
**Bacterial strains**		***glk***	***pgi***	***Pfk-1***	***fbaA***	***tpiA***	***gapA***	***pgk***	***gpmA***	***gpmB***	***eno***	***pykA***	***pykF***	***Zwf***	***pgl***	***edd***	***eda***	***Zwf***	***pgl***	***gnd***	***rpiA***	***tktA***	***rpe***	***tal***	
Ralstonia solanacearum	**CFBP-2957**			■																■					[1]
	**F1C1**			■																■					UP
	**CMR-15**			■																■					[1]
	**Ipo1609**			■																■					[2]
	**K60**			■																■					[3]
	**GMI1000**			■																■					[4]
	**PSI07**			■																■					[1]
	**Po82**			■																■					[5]
	**UY031**			■																■					[6]
	**Y45**			■																■					[5]
	**UW551**			■																■					[3]
	**FQY_4**			■																■					[7]
	**FJAT-1458**			■																■					[8]
	**T78**			■																■					[9]
	**EP1**			■																■					[10]
	**T60**			■																■					[9]
	**SL3882**			■																■					[9]
	**SEPPXO5**			■																■					[11]
	**HA4-1**			■																■					[12]
	**FJAT-91**			■																■					[13]
	**SL3300**			■																■					[9]
	**YC45**			■																■					[14]
	**SL3822**			■																■					[9]
	**CQPS-1**			■																■					[15]

	**OE1-1**																								[16]
	**RSCM**																								[17]
	**SL3755**																								[9]
	**T117**																								[9]
	**T42**																								[9]
	**SL3730**																								[9]
	**SL2729**																								[9]
	**T25**																								[9]
	**SL2330**																								[9]
	**UW386**																								[18]
	**T110**																								[9]
	**T95**																								[9]
	**SL2064**																								[9]
	**KACC10722**																								[19]
	**SL3022**																								[9]
	**SL3175**																								[9]
	**T98**																								[9]
	**T11**																								[9]
	**SL2312**																								[9]
	**T82**																								[9]
	**T101**																								[9]
	**T12**																								[9]
	**T51**																								[9]
	**UW163**																								[20]
	**SL3103**																								[9]
	**IBSBF2570**																								[21]
	**KACC10709**																								[22]

	**RS488**																								[23]
	**RS489**																								[23]
Ralstonia syzygii	**R24**																								[24]
**Banana Blood Disease strain**	**R229**																								[25]
Ralstonia insidiosa	**FC1138**																								[26]
Ralstonia eutropha (Cuprividus nector)	**H16**																								[27]
	**JMP134**																								[28]
Ralstonia pseudosolanacearum	**RS 476**																								[29]
Ralstonia mannitolilytica	**SN82F48**																								[30]
	**12J**																								[31]
Ralstonia pickettii	**12D**																								[32]
	**DTP0602**																								[33]
Escherichia coli	**DH10B**																								[34]

UP: Unpublished data

References:

[1] Remenant B, Coupat-Goutaland B, Guidot A, Cellier G, Wicker E, Allen C, Fegan M, Pruvost O, Elbaz M, Calteau A, Salvignol G, Mornico D, Mangenot S, Barbe V, Médigue C, Prior P. 2010. Genomes of three tomato pathogens within the Ralstonia solanacearum species complex reveal significant evolutionary divergence. BMC Genomics 11:379.

[2] Guidot A, Elbaz M, Carrère S, Siri MI, Pianzzola MJ, Prior P, Boucher C. 2009. Specific Genes from the Potato Brown Rot Strains of *Ralstonia solanacearum* and Their Potential Use for Strain Detection. Phytopathology 99:1105–1112.

[3] Hayes MM, MacIntyre AM, Allen C. 2017. Complete Genome Sequences of the Plant Pathogens Ralstonia solanacearum Type Strain K60 and R. solanacearum Race 3 Biovar 2 Strain UW551. Genome Announc 5.

[4] Salanoubat M, Genin S, Artiguenave F, Gouzy J, Mangenot S, Arlat M, Billault A, Brottier P, Camus JC, Cattolico L, Chandler

M, Choisne N, Claudel-Renard C, Cunnac S, Demange N, Gaspin C, Lavie M, Moisan A, Robert C, Saurin W, Schiex T, Siguier P, Thébault P, Whalen M, Wincker P, Levy M, Weissenbach J, Boucher CA. 2002. Genome sequence of the plant pathogen Ralstonia solanacearum. Nature 415:497–502.

[5] Xu J, Zheng H, Liu L, Pan Z, Prior P, Tang B, Xu J, Zhang H, Tian Q, Zhang L, Feng J. 2011. Complete genome sequence of the plant pathogen Ralstonia solanacearum strain Po82. J Bacteriol 193:4261–2.

[6] Guarischi-Sousa R, Puigvert M, Coll NS, Siri MI, Pianzzola MJ, Valls M, Setubal JC. 2016. Complete genome sequence of the potato pathogen Ralstonia solanacearum UY031. Stand Genomic Sci 11:7.

[7] Cao Y, Tian B, Liu Y, Cai L, Wang H, Lu N, Wang M, Shang S, Luo Z, Shi J. 2013. Genome Sequencing of Ralstonia solanacearum FQY_4, Isolated from a Bacterial Wilt Nursery Used for Breeding Crop Resistance. Genome Announc 1:e00125-13.

[8] Chen D, Liu B, Zhu Y, Wang J, Chen Z, Che J, Zheng X, Chen X. Complete genome sequence of Ralstonia solanacearum FJAT-1458, a potential biocontrol agent for tomato wilt. Genome Announc.. 2017 Apr 6;5(14):e00070-17.

[9] Cho H, Song ES, Lee YK, Lee S, Lee SW, Jo A, Lee BM, Kim JG, Hwang I. Analysis of genetic and pathogenic diversity of Ralstonia solanacearum causing potato bacterial wilt in Korea. The plant pathology journal. 2018 Feb;34(1):23.

[10] Li P, Wang D, Yan J, Zhou J, Deng Y, Jiang Z, Cao B, He Z, Zhang L. Genomic analysis of phylotype I strain EP1 reveals substantial divergence from other strains in the Ralstonia solanacearum species complex. Frontiers in microbiology. 2016 Oct 26;7:1719.

[11] Li X, Huang X, Chen G, Zou L, Wei L, Hua J. Complete genome sequence of the sesame pathogen Ralstonia solanacearum strain SEPPX 05. Genes & genomics. 2018 Jun 1;40(6):657-68.

[12] Tan X, Qiu H, Li F, Cheng D, Zheng X, Wang B, Huang M, Li W, Li Y, Sang K, Song B. Complete genome sequence of sequevar 14M Ralstonia solanacearum strain HA4-1 reveals novel type III effectors acquired through horizontal gene transfer. Frontiers in microbiology. 2019;10:1893.

[13] Sun Y, Wang K, Caceres-Moreno C, Jia W, Chen A, Zhang H, Liu R, Macho AP. Genome sequencing and analysis of Ralstonia solanacearum phylotype I strains FJAT-91, FJAT-452 and FJAT-462 isolated from tomato, eggplant, and chili pepper in China. Standards in genomic sciences. 2017 Dec;12(1):29.

[14] She X, Tang Y, He Z, Lan G. Genome sequencing of Ralstonia solanacearum race 4, biovar 4, and phylotype I, strain YC45, isolated from Rhizoma kaempferiae in southern China. Genome Announc.. 2015 Oct 29;3(5):e01110-15.

[15] Liu Y, Tang Y, Qin X, Yang L, Jiang G, Li S, Ding W. Genome sequencing of Ralstonia solanacearum CQPS-1, a phylotype I strain collected from a highland area with continuous cropping of tobacco. Frontiers in microbiology. 2017 May 31;8:974.

[16] Hikichi, Y., Nakazawa-Nasu, Y., Kitanosono, S., Suzuki, K. And Okuno, T., 1999. The Behavior of Lux-marked Ralstonia, solanacearum in Grafted Tomato Cultivars Resistant or Susceptible to Bacterial Wilt. Japanese Journal of Phytopathology, 65(6), pp.597-603

[17] https://www.ncbi.nlm.nih.gov/bioproject/422474

[18] Swanson, J. K., J. Yao, J. K. Tans-Kersten, and C. Allen. 2005. Behavior of Ralstonia solanacearum race 3 biovar 2 during latent and active infection of geranium. Phytopathology 95:136–143.

[19] https://www.ncbi.nlm.nih.gov/bioproject/314571

[20] Ailloud F, Lowe T, Cellier G, Roche D, Allen C, Prior P. Comparative genomic analysis of Ralstonia solanacearum reveals candidate genes for host specificity. BMC genomics. 2015 Dec;16(1):270.

[21] Rodrigues LM, Destefano SA, Da Silva MJ, Costa GG, Maringoni AC. Characterization of Ralstonia solanacearum strains from Brazil using molecular methods and pathogenicity tests. Journal of plant pathology. 2012 Nov 1;94(3):505-16.

[22] https://www.ncbi.nlm.nih.gov/bioproject/314721

[23] Lopes CA, Mendonca JL. Reação de acessos de jurubeba à murcha bacteriana para uso como porta-enxerto em tomateiro. Horticultura Brasileira 2016; 34: 356–360.

[24] Remenant B, de Cambiaire J-C, Cellier G, Jacobs JM, Mangenot S, Barbe V, Lajus A, Vallenet D, Medigue C, Fegan M, Allen C, Prior P. 2011. Ralstonia syzygii, the Blood Disease Bacterium and some Asian R. solanacearum strains form a single genomic species despite divergent lifestyles. PLoS One 6:e24356.

[25] Remenant B, Coupat-Goutaland B, Guidot A, Cellier G, Wicker E, Allen C, Fegan M, Pruvost O, Elbaz M, Calteau A, Salvignol G, Mornico D, Mangenot S, Barbe V, Médigue C, Prior P. 2010. Genomes of three tomato pathogens within the Ralstonia solanacearum species complex reveal significant evolutionary divergence. BMC Genomics 11:379

[26] Xu Y, Nagy A, Yan X, Haley BJ, Kim SW, Liu NT, Nou X. 2016. Genome Sequences of Ralstonia insidiosa Type Strain ATCC 49129 and Strain FC1138, a Strong Biofilm Producer Isolated from a Fresh-Cut Produce-Processing Plant. Genome Announc 4:e00847-16.

[27] Schwartz E, Henne A, Cramm R, Eitinger T, Friedrich B, Gottschalk G. 2003. Complete Nucleotide Sequence of pHG1: A Ralstonia eutropha H16 Megaplasmid Encoding Key Enzymes of H2-based Lithoautotrophy and Anaerobiosis. J Mol Biol 332:369–383.

[28] Lykidis A, Pérez-Pantoja D, Ledger T, Mavromatis K, Anderson IJ, Ivanova NN, Hooper SD, Lapidus A, Lucas S, González B, Kyrpides NC. 2010. The Complete Multipartite Genome Sequence of Cupriavidus necator JMP134, a Versatile Pollutant Degrader. PLoS One 5:e9729.

[29] Albuquerque GMR, Souza EB, Silva AMF, Lopes CA, Boiteux LS, Fonseca ME de N. 2017. Genome Sequence of Ralstonia pseudosolanacearum Strains with Compatible and Incompatible Interactions with the Major Tomato Resistance Source Hawaii 7996. Genome Announc 5:e00982-17.

[30] Greninger AL, Miller S, CCY. 2015. Ralstonia mannitolilytica. Genome Seq Ralstonia mannitolilytica.

[31] Lucas S, Copeland A, Lapidus A, Glavina del Rio T, Dalin E, Tice H, Bruce D, Goodwin L, Pitluck S, Sims D, Meincke L, Brettin T, Detter JC, Han C, Larimer F, Land M, Hauser L. KNRP. 2009. Ralstonia pickettii (strain 12D). 'Complete Seq genome Ralstonia Pick 12D'.

[32] Ohtsubo Y, Fujita N, Nagata Y, Tsuda M, Iwasaki T, Hatta T. 2013. Complete Genome Sequence of Ralstonia pickettii DTP0602, a 2,4,6-Trichlorophenol Degrader. Genome Announc 1:e00903-13.

[33] Lucas S, Copeland A, Lapidus A, Glavina del Rio T, Dalin E, Tice H, Bruce D, Goodwin L, Pitluck S, Meincke , Brettin T, Detter JC, Han C, Kuske CR, Schmutz J, Larimer F, Land M HLRP. 2008. Ralstonia pickettii (strain 12J). database. EMBL/Genbank/DDBJ.

[34] Durfee T, Nelson R, Baldwin S, Plunkett G, Burland V, Mau B, Petrosino JF, Qin X, Muzny DM, Ayele M, Gibbs RA, Csörgo B, Pósfai G, Weinstock GM, Blattner FR. 2008. The complete genome sequence of Escherichia coli DH10B: insights into the biology of a laboratory workhorse. J Bacteriol 190:2597–606.
