# Supplementary material for: The Entner-Doudoroff and Nonoxidative Pentose Phosphate Pathways Bypass Glycolysis and the Oxidative Pentose Phosphate Pathway in Ralstonia solanacearum
Source: mSystems. 2020 Mar 10;5(2):e00091-20. doi: 10.1128/mSystems.00091-20 (PMC7065512; doi:10.1128/mSystems.00091-20)
Supplement: TABLE S2 [file mSystems.00091-20-st002.pdf]

Supplementary Table S2. Amino acid fragments of *R. solanacearum*. All the detected 15 amino acids with their respective Peak no, elution time (Figure 1), derivative and specific ions (m/z) obtained from GC-MS are tabulated

Peak No	Elution time	Amino acid	Derivative	Specific ions (m/z)			
				[M-85]	Carbon backbone	[M-57]	Carbon backbone
1	12.285	Alanine	2TBDMS	232	2-3	260	1-3
2	12.958	Glycine	2TBDMS	218	2	246	1-2
3	15.622	Valine	2TBDMS	260	2-5	288	1-5
4	16.793	Leucine	2TBDMS	274	2-5	302	1-5
5	17.629	Isoleucine	2TBDMS	274	2-6	302	1-6
6	18.476	Proline	2TBDMS	258	2-5	286	1-5
7	23.632	Methionine	2TBDMS	292	2-5	320	1-5
8	24.262	Serine	3TBDMS	362	2-3	390	1-3
9	24.955	Threonine	3TBDMS	376	2-4	404	1-4
10	26.436	Phenylalanine	2TBDMS	308	2-9	336	1-9
11	28.068	Aspartic acid	3TBDMS	391	2-4	419	1-4
12	30.612	Glutamic acid	3TBDMS	330	2-5	432	1-5
13	32.826	Lysine	3TBDMS	403	2-6	431	1-6
14	36.793	Histidine	3TBDMS	412	2-6	440	1-6
15	37.752	Tyrosine	3TBDMS	438	2-9	466	1-9
