# Supplementary material for: The Entner-Doudoroff and Nonoxidative Pentose Phosphate Pathways Bypass Glycolysis and the Oxidative Pentose Phosphate Pathway in Ralstonia solanacearum
Source: mSystems. 2020 Mar 10;5(2):e00091-20. doi: 10.1128/mSystems.00091-20 (PMC7065512; doi:10.1128/mSystems.00091-20)
Supplement: TABLE S3 [file mSystems.00091-20-st003.pdf]

Supplementary Table S3. Average 13C abundance (in%) of *Ralstonia solanacearum* F1C1 strain (valid, [M-57] & [M-85] amino acids fragment ions and standard deviation from 4 replicates) subjected to minimal media supplemented with [$^{12}C_6$]-, [$^{1\text{-}13}C$]-, [$^{1,2\text{-}13}C$]- and [$^{13}C_6$]-glucose.

Metabolites Fragment	Fragment ion	Carbon numbers	Formula	[$^{12}C_6$]	[1-$^{13}C_6$]	[1,2-$^{13}C_6$]	[$^{13}C_6$]
**Ala-232**	$[M\text{-}85]^+$	2,3	$M - C_5H_9O$	0.6 ± 0.1	1 ± 0.0	29 ± 0.1	36 ± 0.2
**Ala-260**	$[M\text{-}57]^+$	1,2,3	$M\text{-}C_4H_9$	0.2 ± 0.1	20 ± 0.5	41 ± 0.3	37 ± 0.1
**Gly-218**	$[M\text{-}85]^+$	2	$M - C_5H_9O$	0.7 ± 0.3	4 ± 0.5	7 ± 0.2	20 ± 0.3
**Ser390**	$[M\text{-}57]^+$	1,2,3	$M\text{-}C_4H_9$	0.4 ± 0.2	1 ± 0.4	2 ± 0.2	37 ± 0.4
**Ser362**	$[M\text{-}85]^+$	2,3	$M - C_5H_9O$	0.7 ± 0.2	1 ± 0.6	1 ± 0.2	37 ± 0.3
**Val-288**	$[M\text{-}57]^+$	1,2,3,4,5	$M\text{-}C_4H_9$	0.0 ± 0.0	12 ± 0.4	35 ± 0.1	35 ± 0.3
**Val-260**	$[M\text{-}85]^+$	2,3,4,5	$M - C_5H_9O$	0.6 ± 0.1	1 ± 0.1	29 ± 0.1	36 ± 0.3
**Phe336**	$[M\text{-}57]^+$	1,2,3,4,5,6,7,8,9	$M\text{-}C_4H_9$	0.1 ± 0.1	0 ± 0.4	1 ± 0.1	35 ± 0.0
**Phe308**	$[M\text{-}85]^+$	2,3,4,5,6,7,8,9	$M - C_5H_9O$	0.2 ± 0.1	0 ± 0.4	1 ± 0.1	35 ± 0.1
**Tyr466**	$[M\text{-}57]^+$	1,2,3,4,5,6,7,8,9	$M\text{-}C_4H_9$	0.1 ± 0.1	0 ± 0.2	1 ± 0.1	35 ± 0.2
**Tyr438**	$[M\text{-}85]^+$	2,3,4,5,6,7,8,9	$M - C_5H_9O$	0.0 ± 0.0	0 ± 0.2	1 ± 0.2	35 ± 0.1
**His440**	$[M\text{-}57]^+$	1,2,3,4,5,6	$M\text{-}C_4H_9$	0.4 ± 0.1	3 ± 0.3	7 ± 0.2	35 ± 0.3
**His412**	$[M\text{-}85]^+$	2,3,4,5,6	$M - C_5H_9O$	0.3 ± 0.1	4 ± 0.4	8 ± 0.2	35 ± 0.1
