# Supplementary material for: The Entner-Doudoroff and Nonoxidative Pentose Phosphate Pathways Bypass Glycolysis and the Oxidative Pentose Phosphate Pathway in Ralstonia solanacearum
Source: mSystems. 2020 Mar 10;5(2):e00091-20. doi: 10.1128/mSystems.00091-20 (PMC7065512; doi:10.1128/mSystems.00091-20)
Supplement: TABLE S4 [file mSystems.00091-20-st004.pdf]

Supplementary Table S4. The Mass Isotopomer Distributions (MID) of amino acid fragments (valid, [M-57] and/or [M-85] and standard deviation from 4 replicates) of *Ralstonia solanacearum* F1C1 subjected to minimal media supplemented with [$^{12}C_6$]-, [$^{1\text{-}13}C$]-, [$^{1,2\text{-}13}C$]- and [$^{13}C_6$]-glucose

**Metabolite fragment**	**Mass Isotopomers**	**Fragment ion**	**Carbon numbers**	**[$^{12}C_6$]**	**[1-$^{13}C_6$]**	**[1,2-$^{13}C_6$]**	**[$^{13}C_6$]**
Ala232	M0	$[M-85]^+$	2,3	0.9882 ± 0.0025	0.9848 ± 0.0024	0.4269 ± 0.0019	0.5952 ± 0.0019
	M1			0.0112 ± 0.0025	0.0132 ± 0.0020	0.5674 ± 0.0017	0.0807 ± 0.0009
	M2			0.0006 ± 0.0001	0.0020 ± 0.001	0.0057 ± 0.0014	0.3240 ± 0.0015
Ala260	M0	$[M-57]^+$	1,2,3	0.9889 ±0.0036	0.4127 ± 0.013	0.3817 ± 0.0022	0.5804 ± 0.0020
	M1			0.0103 ± 0.0032	0.5856 ± 0.013	0.0262 ± 0.0092	0.0499 ± 0.0017
	M2			0.0008 ± 0.0005	0.0017 ± 0.0005	0.5856 ± 0.0074	0.0592 ± 0.0011
	M3			0.0000 ± 0.0000	0.0000 ± 0.0000	0.0065 ± 0.0015	0.3104 ± 0.0011
Gly218	M0	$[M-85]^+$	2	0.9927 ± 0.0029	0.9626 ± 0.0048	0.9334 ± 0.0018	0.8023 ± 0.0034
	M1			0.0073 ± 0.0029	0.0374 ± 0.0048	0.0666 ± 0.0018	0.1976 ± 0.0034
Ser390	M0	$[M-57]^+$	1,2,3	0.9896 ± 0.0063	0.9818 ± 0.0092	0.9584 ± 0.0045	0.5596 ± 0.0044
	M1			0.0101 ± 0.0060	0.0171 ± 0.0095	0.0379 ± 0.0042	0.0682 ± 0.0032
	M2			0.0003 ± 0.0005	0.0004 ± 0.0006	0.0037 ± 0.0032	0.0781 ± 0.0017
	M3			0.0000 ± 0.0000	0.0007 ± 0.0006	0.0000 ± 0.0000	0.2940 ± 0.0044
Ser362	M0	$[M-85]^+$	2,3	0.9876 ± 0.0038	0.9900 ± 0.0075	0.9777 ± 0.0020	0.5716 ± 0.0030
	M1			0.0114 ± 0.0046	0.0092 ± 0.0065	0.0207 ± 0.0032	0.1268 ± 0.0005
	M2			0.0011 ± 0.0012	0.0007 ± 0.0013	0.0016 ± 0.0024	0.3016 ± 0.0028
Val288	M0	$[M-57]^+$	1,2,3,4,5	1.0000 ± 0.0000	0.4149 ± 0.0187	0.1986 ± 0.0020	0.3855 ± 0.0069
	M1			0.0000 ± 0.0000	0.5851 ± 0.0187	0.2018 ± 0.0026	0.0598 ± 0.0029
	M2			0.0000 ± 0.0000	0.0000 ± 0.0000	0.2397 ± 0.0022	0.2187 ± 0.0030
	M3			0.0000 ± 0.0000	0.0000 ± 0.0000	0.3598 ± 0.0037	0.1913 ± 0.0045
	M4			0.0000 ± 0.0000	0.0000 ± 0.0000	0.0000 ± 0.0000	0.0355 ± 0.0011
	M5			0.0000 ± 0.0000	0.0000 ± 0.0000	0.0000 ± 0.0000	0.1091 ± 0.0003
Val260	M0	$[M-85]^+$	2,3,4,5	0.9774 ± 0.0028	0.9764 ± 0.0045	0.2144 ± 0.0013	0.3739 ± 0.0043
	M1			0.0215 ± 0.0022	0.0167 ± 0.0045	0.4036 ± 0.0011	0.0944 ± 0.0010
	M2			0.0011 ± 0.0011	0.0068 ± 0.0009	0.3742 ± 0.0014	0.3669 ± 0.0024
	M3			0.0000 ± 0.0000	0.0001 ± 0.0001	0.0075 ± 0.0012	0.0534 ± 0.0014
	M4			0.0000 ± 0.0000	0.0000 ± 0.0000	0.0002 ± 0.0001	0.1112 ± 0.0023
Phe336	M0	$[M-57]^+$	1,2,3,4,5,6,7,8,9	0.9925 ± 0.0047	0.9582 ± 0.0049	0.8922 ± 0.0076	0.2108 ± 0.0010

	M1			0.0072 ± 0.0048	0.0406 ± 0.0040	0.0956 ± 0.0084	0.0828 ± 0.0005
	M2			0.0002 ± 0.0004	0.0012 ± 0.0014	0.0117 ± 0.0018	0.1036 ± 0.0007
	M3			0.0000 ± 0.0000	0.0000 ± 0.0000	0.0005 ± 0.0003	0.1935 ± 0.0031
	M4			0.0000 ± 0.0000	0.0000 ± 0.0000	0.0000 ± 0.0000	0.1151 ± 0.0028
	M5			0.0000 ± 0.0000	0.0000 ± 0.0000	0.0000 ± 0.0000	0.1013 ± 0.0018
	M6			0.0000 ± 0.0000	0.0000 ± 0.0000	0.0000 ± 0.0000	0.1036 ± 0.0011
	M7			0.0000 ± 0.0000	0.0000 ± 0.0000	0.0000 ± 0.0000	0.0414 ± 0.0004
	M8			0.0000 ± 0.0000	0.0000 ± 0.0000	0.0000 ± 0.0000	0.0265 ± 0.0004
	M9			0.0000 ± 0.0000	0.0000 ± 0.0000	0.0000 ± 0.0000	0.0212 ± 0.0004
Phe308	M0	$[M-85]^+$	2,3,4,5,6,7,8,9	0.9829 ± 0.0047	0.9689 ± 0.0067	0.9106 ± 0.0031	0.2148 ± 0.0019
	M1			0.0169 ± 0.0049	0.0300 ± 0.0060	0.0852 ± 0.0015	0.0874 ± 0.0007
	M2			0.0000 ± 0.0000	0.0007 ± 0.0008	0.0039 ± 0.0018	0.1787 ± 0.0032
	M3			0.0001 ± 0.0002	0.0002 ± 0.0002	0.0003 ± 0.0003	0.1543 ± 0.0037
	M4			0.0000 ± 0.0000	0.0000 ± 0.0000	0.0000 ± 0.0000	0.1264 ± 0.0015
	M5			0.0000 ± 0.0000	0.0000 ± 0.0000	0.0000 ± 0.0000	0.1126 ± 0.0011
	M6			0.0000 ± 0.0000	0.0000 ± 0.0000	0.0000 ± 0.0000	0.0762 ± 0.0010
	M7			0.0000 ± 0.0000	0.0000 ± 0.0000	0.0000 ± 0.0000	0.0279 ± 0.0009
	M8			0.0000 ± 0.0000	0.0000 ± 0.0000	0.0000 ± 0.0000	0.0217 ± 0.0003
Tyr466	M0	$[M-57]^+$	1,2,3,4,5,6,7,8,9	0.9910 ± 0.0057	0.9721 ± 0.0100	0.9010 ± 0.0040	0.2135 ± 0.0023
	M1			0.0089 ± 0.0057	0.0275 ± 0.0099	0.0889 ± 0.0034	0.0785 ± 0.0029
	M2			0.0000 ± 0.0000	0.0003 ± 0.0003	0.0098 ± 0.0035	0.1058 ± 0.0014
	M3			0.0000 ± 0.0000	0.0000 ± 0.0000	0.0001 ± 0.0002	0.1955 ± 0.0007
	M4			0.0000 ± 0.0000	0.0000 ± 0.0000	0.0000 ± 0.0000	0.1103 ± 0.0026
	M5			0.0000 ± 0.0000	0.0000 ± 0.0000	0.0000 ± 0.0000	0.1043 ± 0.0020
	M6			0.0000 ± 0.0000	0.0000 ± 0.0000	0.0000 ± 0.0000	0.1000 ± 0.0012
	M7			0.0000 ± 0.0000	0.0000 ± 0.0000	0.0000 ± 0.0000	0.0426 ± 0.0010
	M8			0.0000 ± 0.0000	0.0000 ± 0.0000	0.0000 ± 0.0000	0.0277 ± 0.0016
	M9			0.0000 ± 0.0000	0.0000 ± 0.0000	0.0000 ± 0.0000	0.0216 ± 0.0005
Tyr438	M0	$[M-85]^+$	2,3,4,5,6,7,8,9	0.9979 ± 0.0033	0.9858 ± 0.0133	0.9250 ± 0.0159	0.2137 ± 0.0026
	M1			0.0019 ± 0.0033	0.0141 ± 0.0133	0.0749 ± 0.015	0.0880 ± 0.0025
	M2			0.0000 ± 0.0000	0.0000 ± 0.0000	0.0000 ± 0.0000	0.1795 ± 0.0060

	M3			0.0000 ± 0.0000	0.0000 ± 0.0000	0.0000 ± 0.0000	0.1566 ± 0.0021
	M4			0.0000 ± 0.0000	0.0000 ± 0.0000	0.0000 ± 0.0000	0.1239 ± 0.012
	M5			0.0000 ± 0.0000	0.0000 ± 0.0000	0.0000 ± 0.0000	0.1122 ± 0.0057
	M6			0.0000 ± 0.0000	0.0000 ± 0.0000	0.0000 ± 0.0000	0.0757 ± 0.0018
	M7			0.0000 ± 0.0000	0.0000 ± 0.0000	0.0000 ± 0.0000	0.0306 ± 0.0018
	M8			0.0000 ± 0.0000	0.0000 ± 0.0000	0.0000 ± 0.0000	0.0197 ± 0.0019
His440	M0	$[M-57]^+$	1,2,3,4,5,6	0.9764 ± 0.0034	0.8058 ± 0.0161	0.7691 ± 0.0102	0.2709 ± 0.0038
	M1			0.0221 ± 0.0044	0.1911 ± 0.0189	0.0482 ± 0.0111	0.1598 ± 0.0040
	M2			0.0015 ± 0.0020	0.0007 ± 0.0011	0.1776 ± 0.0097	0.1457 ± 0.0032
	M3			0.0000 ± 0.0000	0.0014 ± 0.0012	0.0034 ± 0.0030	0.1915 ± 0.0046
	M4			1E-05 ± 0.0000	0.0004 ± 0.0005	0.0016 ± 0.0011	0.0999 ± 0.0054
	M5			2E-05 ± 0.0000	0.0005 ± 0.0005	0.0000 ± 0.0000	0.0883 ± 0.0016
	M6			0.0000 ± 0.0000	3E-05 ± 0.0000	0.0000 ± 0.0000	0.0436 ± 0.0027
His412	M0	$[M-85]^+$	2,3,4,5,6	0.9921 ±0.0042	0.8052 ± 0.0123	0.7786 ± 0.0088	0.2817 ± 0.0021
	M1			0.0038 ± 0.0032	0.1866 ± 0.0120	0.0478 ± 0.0096	0.1741 ± 0.0062
	M2			0.0025 ± 0.0025	0.0012 ± 0.0022	0.1701 ± 0.0025	0.2532 ± 0.0082
	M3			0.0006 ± 0.0007	0.0060 ± 0.0031	0.0022 ± 0.0017	0.1615 ± 0.0077
	M4			0.0008 ± 0.0020	0.0005 ± 0.0001	0.0008 ± 0.0018	0.0852 ± 0.0028
	M5			0.0002 ± 0.0002	0.0004 ± 0.0008	0.0004 ± 0.0017	0.0440 ± 0.0020
