# Supplementary figures and images for: The Entner-Doudoroff and Nonoxidative Pentose Phosphate Pathways Bypass Glycolysis and the Oxidative Pentose Phosphate Pathway in Ralstonia solanacearum
Source: mSystems. 2020 Mar 10;5(2):e00091-20. doi: 10.1128/mSystems.00091-20 (PMC7065512; doi:10.1128/mSystems.00091-20)

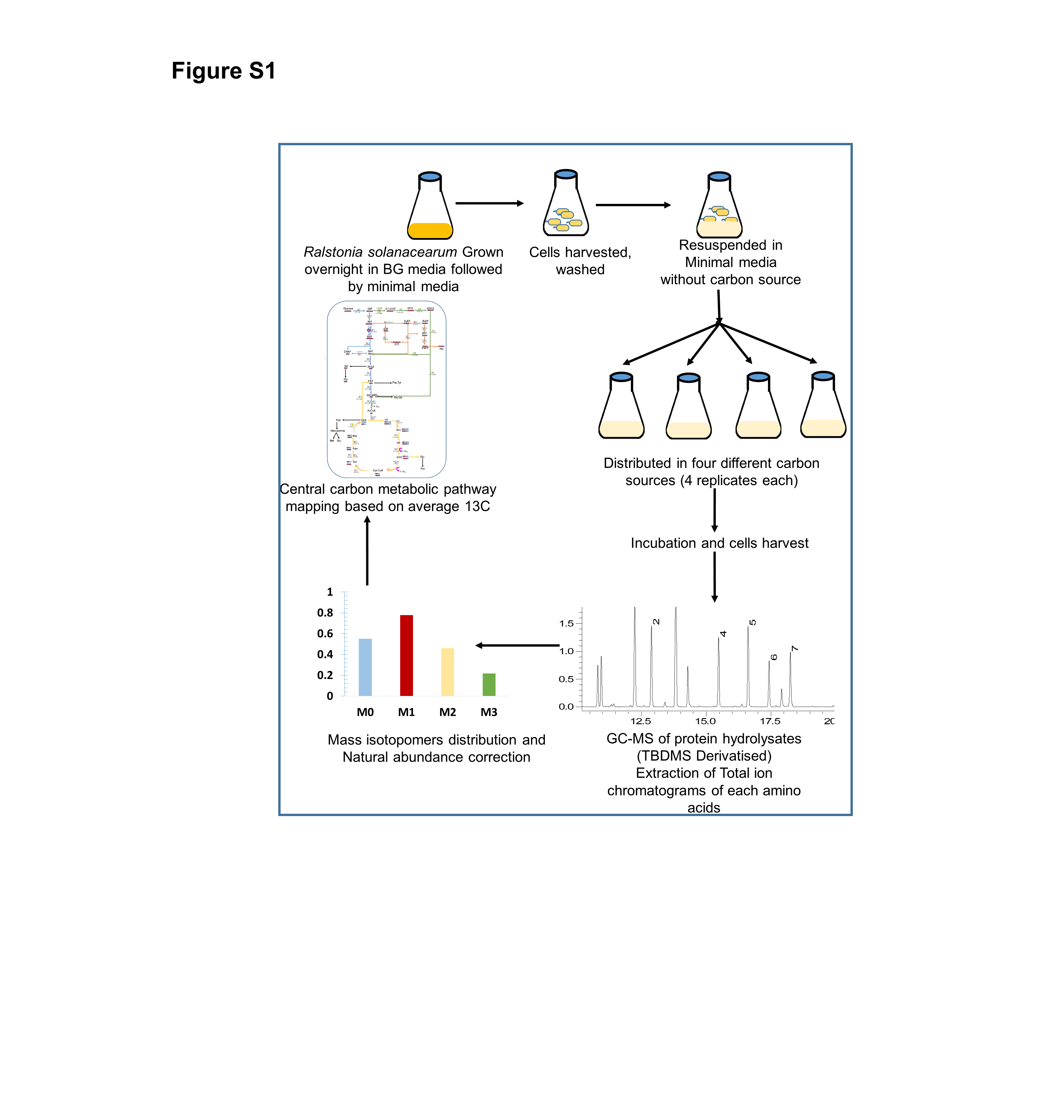

Supplement: FIG S1 [file mSystems.00091-20-sf001.tif]

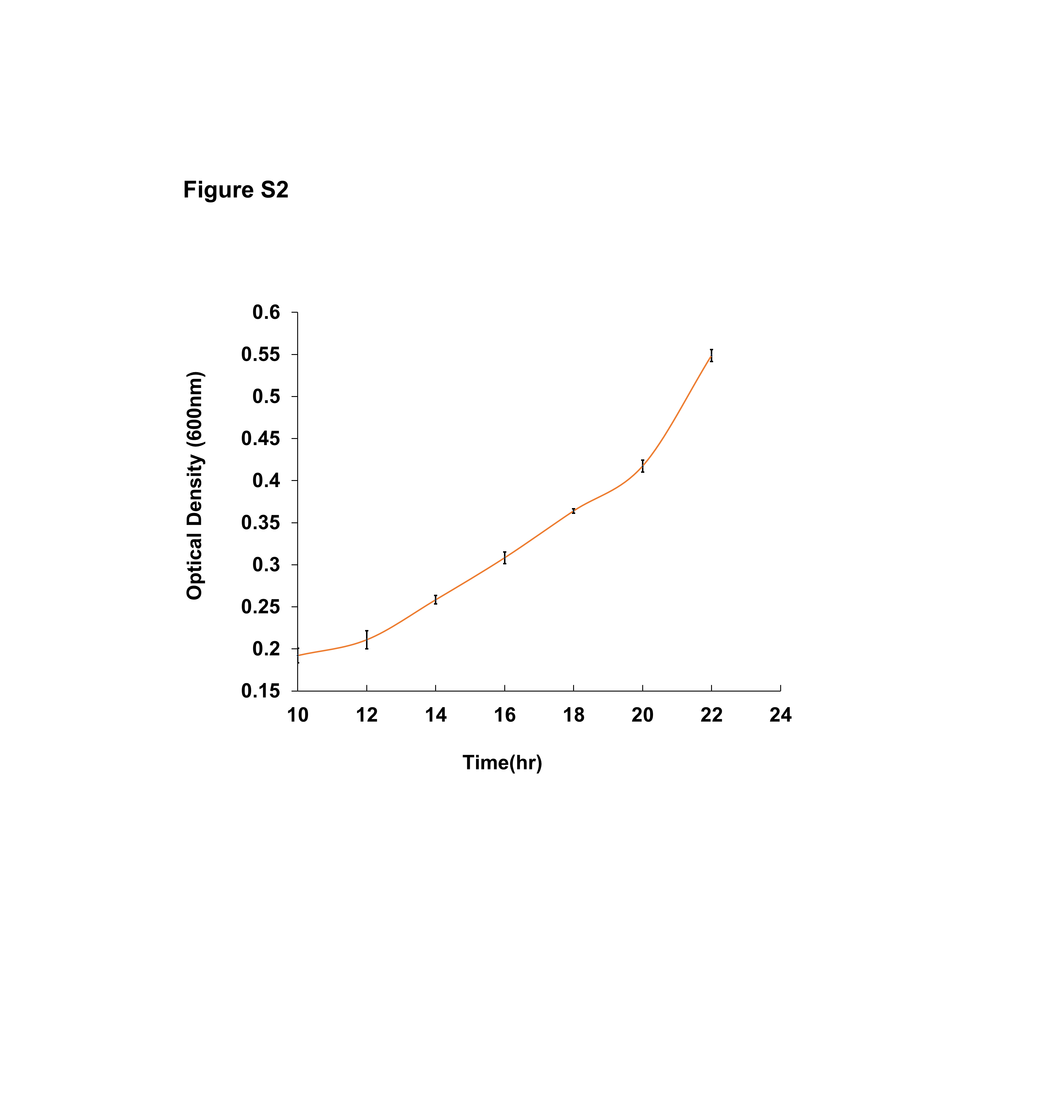

Supplement: FIG S2 [file mSystems.00091-20-sf002.tif]

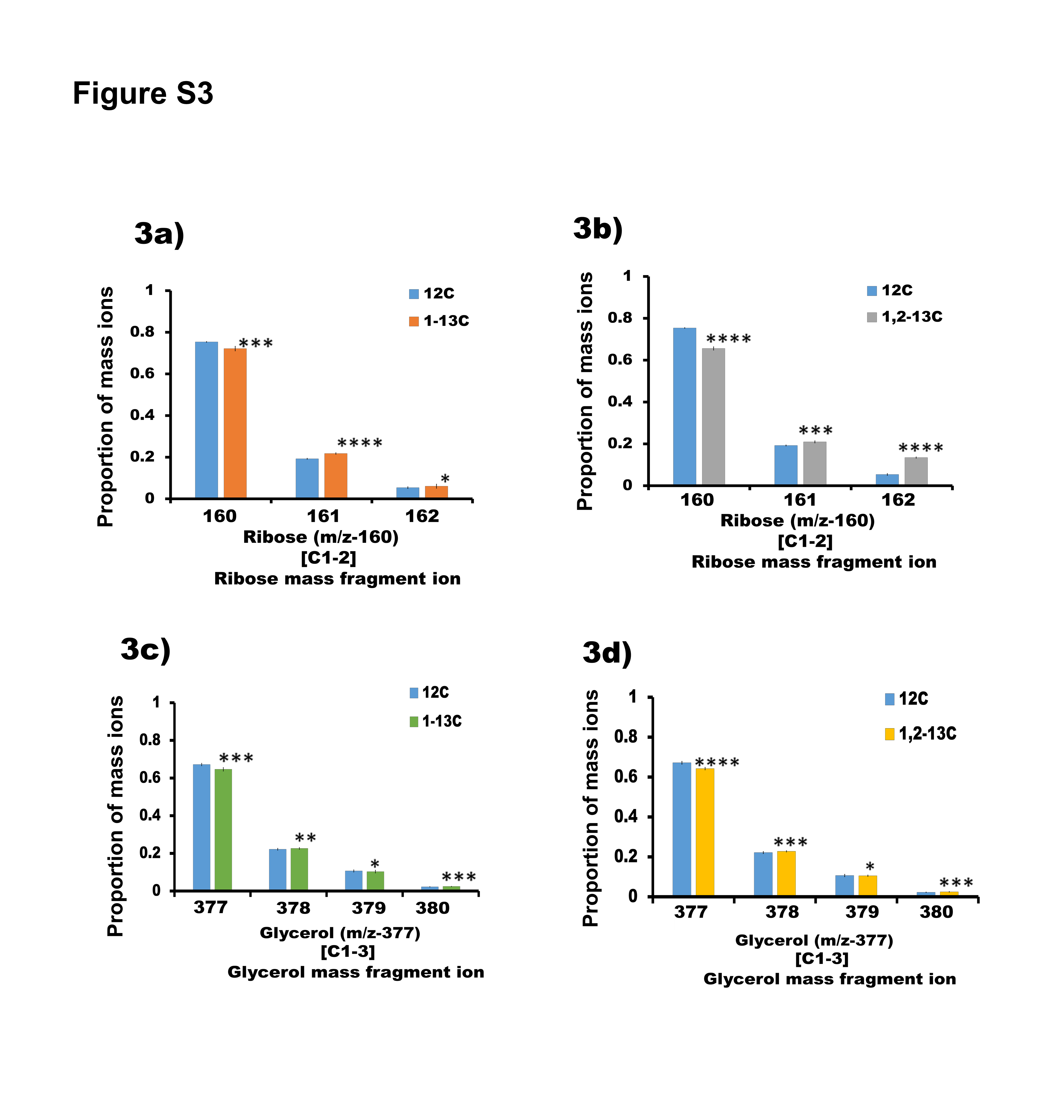

Supplement: FIG S3 [file mSystems.00091-20-sf003.tif]
